# Overcoming the challenge of conducting a pragmatic randomised trial in premises licensed for the on-site sale and consumption of alcohol

**DOI:** 10.1186/1745-6215-14-S1-P28

**Published:** 2013-11-29

**Authors:** Rebecca Playle, Simon Moore, Simon Murphy, Laurence Moore, Kerry Hood, Jonathan Shepherd

**Affiliations:** 1 Cardiff University, Cardiff, UK

## 

A randomised trial was designed to assess the effectiveness and cost effectiveness of the SMILE (Safety Management in Licensed Environments) intervention, delivered to premises licensed for the on-site sale and consumption of alcohol to reduce alcohol-related violence. **Eligible premises were those with identifiable violent incidents on premises, using police recorded violence data.** The intervention is delivered by Environmental Health Officers (EHOs) and outcome data are obtained from the same police recorded violence data during the 12 months following the intervention. The trial design incorporates many methodological challenges. Using routinely collected data for trial purposes involves a great deal of data cleaning and matching to correctly identify both eligible premises for randomisation and incidents related to included premises for outcome analysis. The identification of premises that close either permanently or temporarily in both arms of the trial during follow-up is also an issue. Intervention premises identified as closed during the intervention phase by EHO visits, were replaced from additional eligible premises within the same local authority. However since control premises were not visited in person during the intervention phase it became apparent that we needed to implement a strategy to identify closures in the control arm to assess balance and maintain numbers for comparison in the analysis. The primary analysis will compare the two groups on the number and frequency of violent incidents using the Andersen-Gill model. Identifying the correct intention to treat population in a constantly changing business environment across Wales has been a challenge.

